# Editorial: Magnetoencephalography: Methodological innovation paves the way for scientific discoveries and new clinical applications

**DOI:** 10.3389/fneur.2022.1056301

**Published:** 2022-11-24

**Authors:** Rafeed Alkawadri, Rei Enatsu, Matti Hämäläinen, Anto Bagić

**Affiliations:** ^1^University of Pittsburgh Comprehensive Epilepsy Center (UPCEC), Department of Neurology, University of Pittsburgh Medical Center (UPMC), Pittsburgh, PA, United States; ^2^Department of Neurosurgery, Sapporo Medical University, Sapporo, Japan; ^3^Department of Radiology, Harvard Medical School, Boston, MA, United States; ^4^Department of Neuroscience and Biomedical Engineering, School of Science, Aalto University, Espoo, Finland

**Keywords:** magnetoencephalography, epilepsy, source localization, epilepsy surgery, source estimate, functional mapping, electromagnetism

In 1971, less than five decades after the inception of electroencephalography (EEG), the first real-time magnetoencephalogram was obtained at MIT using a SQUID magnetometer, propelling magnetoencephalography (MEG) as a feasible approach for studying the human brain (1, 2). Then, in 1992, a multidisciplinary research group at the Low-Temperature Laboratory (LTL) of the Helsinki University of Technology (now part of Aalto University) produced the first whole-head MEG system with more than 100 channels (3). The key to this success was the fruitful interactions between the neuroscientists, physicists, mathematicians, engineers, and clinicians who worked together on the instrumentation, analysis methods, and actual neuroscience and clinical applications. Their success reverberated into several research laboratories worldwide, paving the way for MEG to become a recognized method for studying the brain.

During the 21st century, both basic neuroscience and clinical MEG studies have benefited from the use of high-quality open-source academic software packages, which have enhanced the rigor and reproducibility of scientific investigations using MEG. In addition, Optically Pumped Magnetometers (OPMs), novel room-temperature magnetic field sensors, hold promise for significantly improving the spatial resolution and sensitivity of MEG (4). These new devices will also enable the adaptation of the MEG array to the size of the head so that a high signal-to-noise ratio can be achieved, even in studies of early brain development (5). To fully capitalize on these advances, one needs improvements to forward and inverse modeling techniques, as well as to the biophysical models of assemblies of neurons. The latter make it possible to suggest mechanisms underlying the observed macroscopic neural currents, lead to new testable hypotheses, and provide links between recordings in animal models and human MEG (6). Portable and real-time brain-computer MEG-based interfaces will likely become more integrated in the future (2, 7, 8).

The only established clinical applications of MEG, however, are the localization and characterization of epileptic activity (9) and presurgical mapping of the eloquent cortex (10). New studies give hope that MEG, used in combination with EEG and other non-invasive brain imaging methods, will in the future be harnessed for better diagnosis and for monitoring treatment efficacy in several neurological and psychiatric diseases (11, 12).

To that end, MEG has already changed clinical approaches and improved surgical outcomes in epilepsy (13–19), but, paradoxically, it has not yet secured its place in clinical practice (20–23). Furthermore, among the over 20 million patients with drug-resistant epilepsy (DRE) worldwide (2, 24), millions of potential surgical candidates continue to suffer unnecessarily because of the vast underutilization of surgery for epilepsy (2, 15, 25, 26). It appears that the epilepsy community does not have an efficient solution for this cardinal challenge (15, 25–27). Perhaps the blatant lack of synergies between MEG practitioners and the epilepsy community represents an opportunity to change this unfavorable clinical reality; i.e., these two groups could come together to promote non-pharmacologic DRE treatment options and thereby considerably increase the number of comprehensively evaluated patients, including many who could unquestionably benefit from an MEG (9, 23, 28). Yet it seems that previously initiated (i.e., currently stagnant and challenging) efforts to harmonize clinical MEG practice must materialize before we can expect MEG to take its proper place and be used at proper volume in clinical practice (29, 30). Considering that epilepsy surgery is an underutilized tool at large, this possibly applies even more to the underuse of MEG in the context of non-invasive presurgical mapping of the eloquent cortices as part of preparation for surgical interventions (9, 10, 23), where variability in clinical practice may be even greater and the concerted efforts of clinical magnetoencephalographers and neurosurgeons are necessary. In addition to the promise of possible new uses, such as ictal MEG (31, 32), real-world advances have been complicated by logistical concerns, e.g., the duration of recording; monetary, regulatory, or simply practice styles (e.g., handling referrals in less well-established indications such as non-surgical EEG-negative epilepsies); or attitudes toward research (33). However, this has opened doors that allow a more thoughtful approach to applying forward and inverse solutions between old, well-known, and practical ones, like single-point (i.e., single equivalent current dipole) solutions, and perhaps theoretically better and more realistic ones that are already gaining momentum after a slight lag taking advantage of computational and hardware exponential advances. Another ongoing challenge is the lack of a good platform for worldwide data repositories, as well as of consortia that would allow real-time collaboration in an area still practiced in the form of medical art and expert consensus. This is not just a problem with MEG, but with epilepsy surgery in general.

In this collection, we aimed to provide a comprehensive update on the most recent advances in MEG utilization in clinical pre-surgical evaluation, functional mapping, cognitive neuroscience, source localization techniques, and the most recent technological advances. We also highlight network analysis as a newly emerged technique that has approached the pathophysiology of epilepsy from different perspectives. In no particular order: Laohathai et al. discussed fundamental proficiency in the practice of MEG in clinical epilepsy care. Cao et al. presented a perspective on using quantitative network analysis methods for assessing the epileptogenic zone. Sun et al. used magnetoencephalography and graph theory analysis to reveal the dynamics of functional connectivity networks during seizure termination in patients with childhood absence epilepsy. Aung et al. discussed how MEG's excellent temporal and spatial resolutions contribute to the understanding of a subject with both clinical and surgical importance: i.e., what constitutes the boundary between focal, frontal, and generalized epilepsies. Khan et al. reported on different frequency-specific hubs accounting for age-specific maturation. Matsubara et al. discovered that specific functional connectivity was bolstered in patients with benign adult familial myoclonus epilepsy, implying that ipsilateral sensorimotor responses may be a pathologically enhanced motor response homologous to the giant component. Jousmäki offered a unique set of skills and tools that enhance or complement existing commercial solutions with practical mapping applications both in clinical research and in practice. Similarly, Anastasopoulou et al. presented an innovative system that derived kinematic profiles of oro-facial movements during speech, with multiple potential cross-disciplinary applications. Clarke et al. presented a practical approach to addressing noise in data *via* pre-processing and demonstrated it with infant MEG data. Lastly, Mylonas et al. presented a multimodal, non-invasive neurophysiological approach for sleep spindle source localization and discussed its potential clinical applications.

Since its early clinical studies, MEG has provided a non-invasive tool with almost unparalleled temporal and spatial resolutions for various clinical and investigative situations. It has not yet settled in the clinical mainstream, mainly due to the lack of awareness about its indications and potential among practicing physicians, along with its suboptimal representation in the clinical training curricula. This is in addition to the known practical challenges in clinical settings, with their complex and expensive technical prerequisites and environments that are hardly ideal for investigating the true breadth of potential clinical applications. Furthermore, practical implementation of theoretical advances in the software and hardware solutions could potentially replace current, more invasive clinical approaches—for instance, by accurately assessing deep sources and subcortical structures. We believe this journal issue provides a stepping-stone in the right direction to future scientific discoveries and new clinical applications.

## Author contributions

RA, RE, MH, and AB: draft of the manuscript, conceptualization, and revision of the manuscript. All authors contributed to the article and approved the submitted version.

## Conflict of interest

The authors declare that the research was conducted in the absence of any commercial or financial relationships that could be construed as a potential conflict of interest.

## Publisher's note

All claims expressed in this article are solely those of the authors and do not necessarily represent those of their affiliated organizations, or those of the publisher, the editors and the reviewers. Any product that may be evaluated in this article, or claim that may be made by its manufacturer, is not guaranteed or endorsed by the publisher.
